# Correlation between milk suckled and growth of calves of ogaden cattle at one, three and six months of age, east Ethiopia

**DOI:** 10.1186/2193-1801-2-302

**Published:** 2013-07-05

**Authors:** Yesihak Yusuf Mummed

**Affiliations:** School of Animal and Range Science, Haramaya University, Dire Dawa, P.O. Box 138, Ethiopia

**Keywords:** Ogaden cattle, Correlation between milk suckled and growth, Efficiency of milk conversion

## Abstract

This study was conducted with the aim to evaluate correlation between milk suckled and growth of calves of Ogaden cattle managed in Beef farm at Harmaya University, eastern Ethiopia. Data was collected by a method of weight-suckle-weight once in a week from 269 calves born from 1994 to 2004. Weight of calves at birth, first, third and sixth months of age were 21.5 ± 3.3, 36.3 ± 4.4, 57.4 ± 11.0 and 91.7 ± 14.7 kg (mean ± SD). The daily weight gain of calves to first, third and six months of age were 0.5 ± 0.1, 0.4 ± 0.1 and 0.4 ± 0.1 kg day^-1^, respectively. The amount of milk suckled to first, third and six months of age were 5.0 ± 1.2, 4.6 ± 1.1 and 2.7 ± 0.7 kg day^-1^. The milk conversion efficiency to a kg body weight at first and third months of age was 10.2 ± 2.5 and 11.6 ± 2.9, respectively. Correlation between birth weight and daily milk suckled at one, three and six months of age were 0.34 (P< 0.001), 0.22 (P< 0.001) and 0.26 (P<0.05), respectively. The correlation between weight (1 and 3 months) and daily milk suckled at first and third months of age were positive with correlation value of 0.45 (P< 0.001) and 0.43 (P< 0.001), respectively. Correlation between weight change (1 and 3 months) and daily milk suckled at first and third months of age were 0.41 (P< 0.001) and 0.38 (P< 0.001), respectively. Positive association was observed between daily weigh gain and daily milk suckled at first and third months of age with correlation value of 0.44 (P< 0.001) and 0.29 (P< 0.001), respectively. Weight at three months was significantly correlated (P< 0.001) to weight at four, five and six months of age with correlation value of 0.65, 0.63 and 0.53, respectively. The significant correlation between milk suckled and weight at three months; and weight at three months and weight at weaning indicate significant role of milk in determining weaning weight of Ogaden cattle.

## Background

Weaning weight of calves is one of the important traits affecting net income in beef industry. Calf weaning weight is determined by the genetic potential of the calf for growth and the amount of milk received from the dam. Moreover, weight gain to weaning is indicative to maternal ability of the dams. Milk production in beef cattle is difficult to measure there by making any highly correlated trait an important tool in practical selection in beef production. Significant correlation between milk yield and weight gain of calves to weaning was reported for different cattle breed.

Ogaden cattle was reported to produce relatively higher milk yield than the local breed in the region based on the estimate by weight-suckle-weight method (Mummed 2012) and comparable to the yield of the zebu breed based on the estimate by hand milking (Yesihak 2011). The good potential of the breed to be used for beef purpose was reported by Mekuriaw et al. (2009).

Weaning weight is one of the main traits in characterizing cattle. Hence it is important to identify major factors associated with weaning weight. There was no documented information as to the level of association between milk suckled and growth of calves to weaning for Ogaden cattle. The aim of this study was therefore, to evaluate the degree of correlation between milk suckled and growth of calves of Ogaden cattle to the age of six months.

## Result and discussion

### Weight of calves, milk suckled and efficiency of milk conversion

Weight, milk suckled and efficiency of milk conversion of calves of Ogaden Cattle at first, third and sixth months are shown in Table 1. Weight of calves of Ogaden cattle at birth, first, third and sixth months of age were 21.5 ± 3.3, 36.3 ± 4.4, 57.4 ± 11.0 and 91.7 ± 14.7 kg (Mean ± S.D). The weight change to one, three and sixth months of age were 14.8 ± 3.0, 35.8 ± 7.8 and 70.2 ± 15.0 kg. The daily weight gain of the calves to one, three and six months of age were 0.5 ± 0.1, 0.4 ± 0.1 and 0.4 ± 0.1 kg day^-1^, respectively. The amount of daily milk suckled to one, three and six months of age were 5.0 ± 1.2, 4.6 ± 1.1and 2.7 ± 0.7 kg day^-1^. The milk conversion efficiency to one and three months of age were and 10.2 ± 2.5, 11.6 ± 2.9 kg milk for a kg of body weight gain, respectively. The average birth and one month weight of Ogaden calves in the current study was comparable to Fogera breed managed in research station under pasture condition which was 22 kg and 35.1 kg, respectively. However, the weights at three and six months of the breed were relatively higher than the report for Fogera breed which was 49.8 and 68.2 kg, respectively (Bitew et al. 2010). The weaning weight of Ogaden cattle breed in this study was comparable to the report for Horro, Barca and Boran breed at research centers managed under pasture condition in Ethiopia which were 88, 92 and 95 kg, respectively (Demeke et al. 2003). Moreover, the average daily weight gain to weaning was comparable for Horro, Barca and Boran which were 377.6, 385.3 and 401.4 gm, respectively (Demeke et al. 2003). However the average daily weight gain to six months of age in this study was relatively more than what was reported for Fogera breed which was 0.321 kg (Bitew et al. 2010).

**Table 1:** **Weight**, **daily milk suckled and efficiency of milk conversion of calves of Ogaden Cattle** (**Mean** ± **S**.**D**)

Age and weight of calves	Mean	S.D
Birth weight	21.5	3.3
One month		
Weight (kg)	36.3	4.4
Weight change (kg)	14.8	3.0
DWG (kg)	0.5	0.1
DMY (kg)	5.0	1.2
DMY/DWG	10.2	2.5
Three months		
Weight (kg)	57.4	11.0
Weight change (kg)	35.8	7.8
DWG (kg)	0.4	0.1
DMY (kg)	4.6	1.1
DMY/DWG	11.6	2.9
Six months		
Weight (kg)	91.7	14.7
Weight change (kg)	70.2	15.0
DWG (kg)	0.4	0.1
DMY (kg)	2.7	0.7
DMY/DWG	-	-

*DMY* daily milk yield, *DWG* daily weight gain, *DMY/DWG* efficiency of milk conversion, *S.D* standard deviation.

The amount of milk suckled by calves was relatively higher in the first and third months and relatively lower in the six months of age of calves in this study. The higher milk suckled in earlier age could be due to the more dependence of the calves on milk as the rumen was not well developed to take solid feed at early age of its life. In this study, the calves suckled 14%, 8% and 3% of their body weight in the first, third and sixth months of ages, respectively. The milk suckled per kg body weight to three months of age was in acceptable range compared to the general principle of feeding milk to calves about 10% of its body weight to the age of three months. The milk conversion efficiency of the calves in the first and third months of age was 10.2 ± 2.5 and 11.6 ± 2.9, respectively. Higher milk conversion efficiency was observed for calves in the first than the third months of age.

### Correlation between weight of calves and milk suckled

Correlation between weights of calves and milk suckled is shown in Table 2. Birth weight was positively correlated with daily milk suckled (at 1, 3 and 6 months), efficiency of milk conversion (at 1 and 3 months) and weight (at 1 and 3 months) of age. However, there were no significant correlation between birth weight with daily weight gain and with weight change at first, third and sixth months of age. The correlation between birth weight and weight at first month was stronger (0.74) which become moderate at three months (0.43) and weaken at six months (0.21) of age. The magnitude of these value indicated that heavier calves at birth were able to maintain their weight in advantage to weaning weight. Similar to the present finding, the correlation between birth weight and weaning weight was reported 0.30 for Herford breed (Mwansa et al. 2002). However, the correlation between the two traits in this study was relatively lower than correlation value of 0.58 reported for N’Dama breed in the tropic of Nigeria (Abdullah and Olutogun 2006). The association between birth weight and efficiency of milk conversion was moderate in the first and third months of age and was not significant at six months. This indicated the ability of heavier calves at birth to efficiently convert milk to body weight up to three months of age. However, since the effects of birth weight reduce as the calves advance in age, the association between the two traits almost disappeared at six months of age. The absence of significant and strong correlation between birth weight and daily weight gain in this study indicated that birth weight is not good criteria to use in selection of Ogaden cattle for subsequent weight gain. However, it can be used to select weight at first, third and six months of age as the two traits correlated significantly in these ages.

**Table 2:** **Correlation between weights and milk suckled by calves of Ogaden cattle**

	Age of calve	DMS	DWG	DMS/DWG	Weight change	Weight
Birth weight	1	0.34	0.12	0.25	0.15	0.74
		0.00	0.26	0.02	0.16	0.00
	3	0.22	0.11	0.24	0.05	0.43
		0.00	0.31	0.00	0.50	0.00
	6	0.26	0.19	0.09	-0.09	0.21
		0.01	0.08	0.40	0.43	0.05
Weight at Month	1	0.45	0.89	-0.34	0.45	
		0.00	0.00	0.00	0.00	
	3	0.43	0.87	-0.31	0.92	
		0.00	0.00	0.00	0.00	
	6	0.19	0.80	-0.49	0.96	
		0.07	0.00	0.00	0.00	
Age (days)	1	0.11	-0.33	0.33		
		0.28	0.00	0.00		
	3	0.01	-0.17	0.12		
		0.90	0.01	0.08		
	6	-0.42	-0.26	-0.18		
		0.00	0.02	0.09		
Weight change	1	0.41	0.98	-0.55		
		0.00	0.00	0.00		
	3	0.38	0.96	-0.45		
		0.00	0.00	0.00		
	6	0.12	0.76	-0.53		
		0.28	0.00	0.00		
DMS/DWG	1	0.61	-0.47			
		0.00	0.00			
	3	0.58	-0.48			
		0.00	0.00			
	6	0.61	-0.44			
		0.00	0.00			
DWG	1	0.44				
		0.00				
	3	0.29				
		0.00				
	6	0.13				
		0.14				

*DMS* daily milk suckled, *DWG* daily weight gain, *DMS/DWG* efficiency of milk conversion.

The weights at the first and the third months were positively correlated with daily milk suckled at first and third months of age. Moreover, weight at 1st, 3rd and 6th months was positively correlated with daily weight gain and weight change while negatively correlated with efficiency of milk conversion. The correlation value between weights (at 1 and 3 months) and daily milk suckled was moderate at the first and the third months (0.45 and 0.43, respectively) and non significant at six months of age. Similar to the present finding, moderate correlation value between the two traits was reported for Fogera cattle breed managed in research station under pasture condition in Ethiopia (Bitew et al. 2010). The correlation value between weight at one, three and sixth months of age with daily weight gain was between 0.80 and 0.89 in this study. Similar to the present finding, a correlation value of 0.99 was reported between weaning weight and daily weight gain for N’Dama breed in the tropic of Nigeria (Abdullah and Olutogun 2006). The correlation between weights at consecutive age and milk conversion efficiency was moderate and negative in present study.

The correlation between age of calves and daily weight gain was negative in this study. The negative correlation value between the two traits indicated the decrease in daily weight gain as the calves advanced in age. This can be explained by the general fact that the growth rate of animal reduces as they advances in age. The correlation between age of calves at one month and the efficiency of milk conversion was moderate and positive. This further confirmed the higher efficiency of calves in milk conversion at one month of age.

Weight changes were positively correlated with daily milk suckled at one and three months of age, daily weight gain in all age but negatively correlated with efficiency of milk conversion in all categories of age. The positive moderate correlation between weight change of calves and daily milk suckled at one (0.41; P<0.001) and three (0.38; P<0.001) months of age indicated that the change in weight might be partially attributed to daily milk suckled in these age categories. However, the non significant correlation between the two factors at six months of age indicated the possible contribution of other factors than liquid milk for weight change in this stage of life. The negative correlation between weight change and efficiency of milk conversion could be due to the inverse relationship between efficiency and daily weight gain as the daily weight gain directly and strongly correlated with weight change.

Efficiency of milk conversion was positively correlated (P< 0.001) with daily milk suckled and negatively correlated (P< 0.001) with daily weight gain in consecutive ages. The positive correlation between efficiency and milk suckled indicated that calves consumed more milk were efficient in milk conversion. However, the negative correlation (-0.47, -0.48 and -0.44, respectively) between milk conversion efficiency and daily weight gain can be explained by the presence of the inverse relationship between the two traits.

There was significant positive correlation between daily weight gain and daily milk suckled at one (0.44, P< 0.001) and three (0.29, P< 0.001) months of age. The moderate correlations between the two traits at one month weaken in the third month and disappeared in six months. The reduction in degree of correlation between the two traits as the calved advanced in age further confirms the importance of milk in early age which reduced as the animal advanced in age. The association between the two traits was similarly reported for Fogera cattle breed in research station under pasture condition (Bitew et al. 2010).

### Correlation between weights of calves from one to six months of age

Correlation between weights of calves from one to six months of age is shown in Table 3.

**Table 3:** **Correlation between weights of calves from one to six months of age**

Weight	Month1	Month2	Month3	Month4	Month5
Month2	0.66				
	0.00				
Month3	0.49	0.56			
	0.00	0.00			
Month4	0.30	0.57	0.65		
	0.02	0.00	0.00		
Month5	0.21	0.39	0.63	0.83	
	0.11	0.00	0.00	0.00	
Month6	0.11	0.36	0.53	0.74	0.86
	0.41	0.00	0.00	0.00	0.00

Weight at first month of age was significantly associated with weight at second, third and fourth months of age with correlation value of 0.66 (P< 0.001), 0.49 (P< 0.001) and 0.30(P< 0.05), respectively. Significant correlation value between first and second months of age was similarly reported for Brangus cattle which were 0.62 (De Torre and Rankin 1978). In the present study, weight at third months of age was significantly (P< 0.001) correlated with weight at fourth, fifth and sixth months of age with correlation value of 0.65, 0.63 and 0.53, respectively. The correlation between weights at consecutive age indicated that heavier calves in the early age remain heavier throughout the remainder of their growth phase.

## Conclusions

From the study it was concluded that there was significant correlation between birth weight and milk suckled, efficiency of milk conversion and weight at the first and the third months of age. Moreover, at one and three months of age there was significant correlation between daily milk suckled and daily weight gain, weight change, weight at specific age of calves. However, there was no significant correlation between milk suckled and these traits at six months of age. Weight at three months was significantly correlated with weight at fourth, fifth and sixth months. The significant correlation between milk suckled and weight at three months; and weight at three months and weight at weaning indicate significant role of milk in determining weaning weight of Ogaden cattle.

## Methods

### Description of study area

The study was conducted at Haramaya University, Ethiopia. The site is located at 09°N and 42°E at an altitude of 1980 m above sea level. The area receives annual average rainfall of 790 mm and annual mean temperature of 16°C (Mishra et al. 2004).

### Management of experimental animals

Pure Ogaden cattle breed in Beef farm at Haramaya University was used for this study. The breed was described as a strain of Boran breeds (Rege and Tawah 1999). It is the dominant breed in south eastern region of Ethiopia. The farm was established with the objective of characterizing the breed. The farm accommodated 180 – 300 head of cattle. Animals on the farm grazed natural pasture for about eight hours per day and had free access for water. The pasture in the area dominant by *Hyparrhenia* species, *Cynodon dactylon*, *Sporobolus aficanus* and *pennisetum* species (Mengistu and Asnakeh 1986). During the dry season the herd was supplemented with grass hay and occasionally with concentrate. Milk from cows was exclusively left for the calves. Calves were allowed to suckle their dams without restriction up to weaning age. Calves were weaned in batch at about six months of age. Calves had free access to green pasture starting from third weeks of age.

Natural controlled breeding system was practiced on the farm. Bulls were allowed to run with cows during breeding season which usually last for about two months. One breeding bull was used for 20 to 25 breeding cows. Selection of breeding bulls was made based on weight at birth, weaning and yearling. Heifers were allowed to breed when they attained a body weight about 200 kg.

Preventive and control measures were taken against major diseases in the region. Vaccination was given regularly against Anthrax, Black leg, Pasteurellosis, Contagious Bovine Pleuropneumonia and Rinderpest. The herd was dewormed and sprayed against gastro intestinal parasites and external parasite regularly.

Data on growth performance and milk yield of the breed were recorded regularly since the establishment of the farm. Calves born on the farm weighted at birth and once in a week up to the age of weaning. Then after, they were weighted every other week until culled from the farm. Date of mating and calving were properly recorded on recording sheet. Milk yield of cows on the farm was measured indirectly once in a week by a method of weigh-suckle-weigh.

### Data collection

Data recorded on milk yield of the cows by method of weigh-suckle-weigh between 1994 and 2004 was used for the study. A total of 269 cows-calves pair were used for the purpose. In this method, the amount of milk suckled by calves was measured once in a week from birth to sixth months of weaning age. Data was collected after separating the dam and calves from 5 PM to 7 AM. During this activity, each calf was weighted before and after being allowed to suckle their dams to satiety. Differences between pre and post suckling weight were recorded as estimated morning (AM) milk suckled. AM milk suckled was converted to a 24 hr yield based on the formula ([Milk weight/14 hr] × 24 hr) to get predicted daily milk suckled (Brown et al. 1996). The same data collected by weight suckle weigh method was used to generate weight at different age of calves.

### Statistical analysis

Correlation between daily milk suckled (DMS), birth weight, daily weight gain (DWG), weight change, weight at first, third and six months of age and efficiency of milk conversion (DMS/DWG) were measured using Pearson correlations coefficient in Minitab software release 12.21 (Minitab 1998).

Weight change was calculated as the difference between weight at specific month and birth weight. Daily weight gain (DWG) was calculated as the ration of weight change to numbers of days to specific age. Efficiency of milk conversion was calculated as the ratio of daily milk suckled and daily weight gain at the specific age.

## Abbreviations

SD: Standard deviation
DMS: Daily milk Suckled
DWG: Daily weight gain
DMS/DWG: Efficiency of milk conversion.
